# Supplementary material for: Acceptability and perceived facilitators and barriers to the usability of biometric registration among infants and children in Manhiça district, Mozambique: A qualitative study
Source: PLoS One. 2021 Dec 17;16(12):e0260631. doi: 10.1371/journal.pone.0260631 (PMC8683034; doi:10.1371/journal.pone.0260631)
Supplement: S1 Appendix — (DOC) [file pone.0260631.s001.doc]

|  | **Centro de Investigação em Saúde de Manhiça**  **Guião de Entrevista Semi-estruturada (SSI) para profissionais de saúde**  **Versão 1 de 29 de Abril de 2019** | Data da entrevista  ____/ _____/ ______/ |
| --- | --- | --- |

Recolha de dados biométricos em bebês e crianças moçambicanas: avaliação de um aparelho biométrico infantil em fase de testes (protótipo) para aferir com precisão uma única identidade.

**Título abreviado:**

Um estudo para determinar a adequação e estabilidade da biometria em neonatos, bebés e crianças no distrito de Manhiça,

Moçambique

**(Projecto BioNIC)**

Chamo-me ________ do Centro de Investigação em Saúde da Manhiça, gostaria de lhe dar boas-vindas a esta entrevista. Eu serei o facilitador/a desta entrevista e peço para gravar de modo a não perder a informação importante que vai dar ao longo da conversa e caso não aceite ser gravada peço para tomar notas ao longo da entrevista. Toda a informação gravada ou registada será confidencial e não será publicada com o seu nome.

**Propósito**

Esta entrevista semi-estruturada têm como objectivo explorar as percepções dos profissionais de saúde em relação ao uso do dispositivo biométrico infantil na unidade sanitária para fins de recolha de dados biométricos em bebés e crianças dos 0-4 anos de idade. Foi convidado (a) a participar desta entrevista que durará aproximadamente 40 minutos porque a tua contribuição é importante porque nos ajudará a entender se uso do dispositivo biométrico infantil é aceitável ou não no distrito de Manhiça bem como a entender as barreiras e facilitadores que podem influenciar a aceitação desse dispositivo.

**Informações sobre a ocorrência da entrevista**

| ID do entrevistador: |
| --- |
| Foi lido ao participante um consentimento informado?  SIM  NÃO |
| Em que língua o consentimento informado foi explicado  Changana  Português |
| O consentimento informado foi assinado?  SIM  NÃO |
| O consentimento foi assinado antes das perguntas?  SIM  NÃO |
| Data de assinatura do Consentimento Informado: \|D\|D\| - \|M\|M\| - \|A\|A\|A\|A\| |
| Iniciais da pessoa que explicou o consentimento informado: \|__\|__\|__\| |
| O entrevistado aceita gravar a conversa em áudio durante a entrevista?  SIM  NÃO |
| A entrevista foi gravada em áudio?  SIM  NÃO  Se não Razões_________________________________________________________________________________________________________ |
| Local onde decorreu a entrevista: Gabinete do hospital Corredor do hospital Encasa Outro, especifique:______________ |
| Resultado da entrevista: \|__\| Completa \|__\| Incompleta |
| Breve historial da entrevista: |
| Nome da unidade sanitária onde o participante trabalha____________________________  Idade do participante:­­­­­____________  Sexo  Feminino  Masculino  Nível de escolaridade:___________  Profissão do participante__________________________ Tipo de educação / treinamento clinico recebido (por exemplo, médico, técnico, enfermeiro):  Tempo que trabalha nesta unidade sanitária_______________ |

| **Identificação das crianças no sistema de saúde**   1. Pode nos falar sobre a recolha de informação para identificação dos pacientes (crianças) nesta unidade sanitária? 2. Já teve alguma dificuldade para identificar crianças que atendem esta unidade sanitária? 3. Se sim, quais são os desafios ou problemas que os profissionais de saúde encontram no processo de identificação das crianças? 4. As dificuldades de identificação das crianças afectam na qualidade de dados e na prestação dos serviços de saúde? Porquê? 5. Quais são os riscos de não fazer uma identificação correcta da criança para a própria criança e para o sector de saúde?   **Uso de tecnologia biométrica infantil**   1. Na sua opinião qual seria a melhor idade para recolha de informação biométrica em crianças? 2. Acha que o uso de identificação biométrica infantil pode ser uma boa solução para a identificação das crianças na unidade sanitária? 3. Acha que as enfermeiras poderiam fazer o registo dessa identificação no futuro usando os telefones e tablets?   **Conhecimento do estudo sobre Biometria Infantil**   1. Você conhece o estudo BioNic? Se sim, explore em que consiste? Se não, conhece explique ao participante sobre o estudo. 2. Terá visto alguém nesta unidade sanitária a recolher dados biométricos (imagens) de recém-nascidos e crianças de 0-4 anos de idade? Se SIM, fez para que finalidade? Quando é que decorreu a recolha dessas imagens? 3. Você já tinha visto a recolha de imagens de palmas das mãos, dos pés e de orelhas das crianças dos 0-4 anos para fins de identificação biométrica? Se sim, explora onde viu? E quem eram as pessoas que tiravam as imagens? 4. Qual é a sua opinião em relação a recolha de imagens de palmas das mãos, dos pés e de orelhas das crianças dos 0-4 anos para fins de identificação biométrica?      1. Qual é a sua opinião em relação ao uso do dispositivo biométrico infantil? (Sondar: O que você gosta nesta aplicação? O que você não gosta (uso, teclados, camera, tela ou manuseamento)? 2. Na sua opinião quais seriam as vantagens e desvantagens de usar fotografias de palmas das mãos, dos pés e de orelhas das crianças dos 0-4 anos para fins de identificação? 3. Além do estudo BioNic, existe algum sector desta unidade sanitária que captura imagens de crianças dos 0-4 anos de idade para algum fim? [Sondar] Se sim, qual é esse sector? Que dispositivo é usado para a captura das imagens? E que parte do corpo são capturadas as imagens? E para que fim são recolhidas as imagens?     **Viabilidade e aceitabilidade do dispositivo biométrico**   1. Na sua opinião o uso do dispositivo biométrico como actividade de rotina na unidade sanitária pode criar impacto nos cuidados de saúde de rotina nesta Unidade Sanitária? Porquê? 2. Na sua opinião o que pode levar os pais ou encarregados de educação a não aceitarem que as suas crianças dos 0-4 anos de idade sejam retiradas imagens para a sua identificação biométrica? 3. O que poderá ser feito para que os pais aderem sem qualquer constrangimento ao processo de registo e uso de informação biométrica das crianças nas unidades sanitárias? 4. Acha que as mães que foram solicitadas pelos inquiridores para recolha de imagens (palmas das mãos, dos pés e orelhas) das suas crianças dos 0-4 anos de idade aceitavam com facilidade participar do estudo? Porquê? |  |
| --- | --- |

**Fim da entrevista (SSI)**

Comentários Finais:

Estamos muito gratos por você ter concordado em participar desta entrevista. Sabemos que ocupamos o teu tempo precioso. Os teus comentários são muito importantes parque irão nos ajudar a avaliar a aceitabilidade dos pais em relação a recolha de dados biométricos nas suas crianças para efeitos de identificação.

Comentários

**________________________________________________________________________________________________________________________________________________________________________________________________________________________________________________________________________________________________________________________________________________________________________________________________________________________________________________________________________________________________________________________________________________________________________________________________________________________________________________________________________________________________________________________**
