# Supplementary material for: Acceptability and perceived facilitators and barriers to the usability of biometric registration among infants and children in Manhiça district, Mozambique: A qualitative study
Source: PLoS One. 2021 Dec 17;16(12):e0260631. doi: 10.1371/journal.pone.0260631 (PMC8683034; doi:10.1371/journal.pone.0260631)
Supplement: S2 Appendix — (DOC) [file pone.0260631.s002.doc]

**Departamento de ciências sociais do CISM**

Recolha de dados biométricos em bebês e crianças moçambicanas:

Avaliação de aparelho biométrico infantil em fase de testes (protótipo) para aferir com precisão uma única identidade.

Título abreviado:

Um Estudo para Determinar a Adequação e Estabilidade da Biometria em Neonatos, Bebés e Crianças

no Distrito de Manhiça, Moçambique (Projecto BioNIC)

Guião de Discussão em Grupo Focal (DGF

Pais e tutores de crianças residentes na Macia e que não foram recrutadas para participar na recolha de dados biométricos

Chamo-me ___________________ do Centro de Investigação em Saúde da Manhiça, e gostaria de lhes dar as boas-vindas a esta discussão. Eu serei o facilitador desta DGF e estará comigo o Sr/Sra. _______________ também do Centro de Investigação em Saúde da Manhiça que vai tomar notas e gravar esta discussão mediante a vossa permissão.

**Propósito**

Esta discussão em Grupo Focal (DGF) tem como objectivo recolher dados que nos permitirão avaliar a aceitabilidade das mães das crianças dos 0 a 4 anos de idade em relação ao uso do aplicativo de um telemóvel para retirada de fotografias de orelhas, palmas das mãos e dos pés das suas crianças para permitir que sejam identificadas com precisão quando forem a unidade sanitária sem o cartão ou bilhete de identidade da criança. A vossa participação e contribuição é importante. Por isso foram convidados para participar desta discussão em grupo focal (DGF) porque podem fornecer informações que nos permitirão avaliar a usabilidade da biometria infantil e identificar facilidades e dificuldades para o seu uso como forma de garantir uma melhor utilização da identificação única de bebes e crianças. A duração desta discussão em grupo focal está prevista para 60 á 90 minutos.

**Regras Básicas**

Estaremos gravando esta discussão em grupo focal para garantir que nenhuma das respostas que vocês derem não se perca. O meu colega redactor estará também a tomar notas durante a discussão. Todas as informações registadas serão mantidas em sigilo e não serão identificados pelo vosso nome. Vocês podem optar por não responder a qualquer momento.

A fim de promover a coesão do grupo e dar a todos oportunidade igual para falar, vamos seguir as seguintes regras do grupo:

• Todos os participantes terão a oportunidade de responder, se o desejarem;

• Todos os participantes vão esperar pela sua vez para falar;

• Todos os participantes vão respeitar o ponto de vista do outro;

1. **Informação demográfica das participantes**

| Nr | Sexo | Idade | Estado civil | Nível de Escolaridade | Sabe ler | Sabe escrever | Ocupação | Religião | Bairro | Quantos anos tem o teu último filho |
| --- | --- | --- | --- | --- | --- | --- | --- | --- | --- | --- |
| 1 |  |  |  |  |  |  |  |  |  |  |
| 2 |  |  |  |  |  |  |  |  |  |  |
| 3 |  |  |  |  |  |  |  |  |  |  |
| 4 |  |  |  |  |  |  |  |  |  |  |
| 5 |  |  |  |  |  |  |  |  |  |  |
| 6 |  |  |  |  |  |  |  |  |  |  |
| 7 |  |  |  |  |  |  |  |  |  |  |
| 8 |  |  |  |  |  |  |  |  |  |  |
| 9 |  |  |  |  |  |  |  |  |  |  |
| 10 |  |  |  |  |  |  |  |  |  |  |
| 11 |  |  |  |  |  |  |  |  |  |  |
| 12 |  |  |  |  |  |  |  |  |  |  |
|  | | | | | | | | | | |
| 1. **Informações sobre a ocorrência da entrevista** | | | | | | | | | | |
| **Referência do ficheiro: BioNIC_MZ_F1_DGF_** | | | | | | | | | | |
| ID do facilitador: | | | | | | | | | | |
| ID do redactor: | | | | | | | | | | |
| Local da realização da DGF: | | | | | | | | | | |
| Data da realização da DGF: | | | | | | | | | | |
| Número inicial dos participantes da DGF: | | | | | | | | | | |
| Número final dos participantes da DGF: | | | | | | | | | | |
| Hora do início da DGF: | | | | | | | | | | |
| Hora final da DGF: | | | | | | | | | | |
| Língua (s) falada (s) durante a entrevista: | | | | | | | | | | |
| A DGF foi gravada: | | | | | | | | | | |
| Breve Descrição dos Participantes: | | | | | | | | | | |
| Resultado da DGF: |__|Completo |__|Incompleto  Razões: __________________________________________________________________________________________________  _________________________________________________________________________________________________________ | | | | | | | | | | |

3. Perguntas para as participantes

| 1. Você acha aceitável que sejam tiradas fotografias de crianças para fins de pesquisa? 2. Você acha aceitável que as fotografias sejam usadas para identificação? 3. Na sua opinião quais seriam os perigos / vantagens de usar fotografias para a identificação? 4. Qual é parte do corpo que é aceitável / inaceitável para se usar em uma biometria? | **RESUMO** |
| --- | --- |

**Fim da DGF!**

**Comentários Finais:**

**Comentários finais:**

Estamos muito gratos por vocês terem concordado em participar desta importante discussão. Sabemos que ocupamos o vosso precioso tempo. As vossas contribuições foram muito importantes e irão nos orientar na implementação da identificação única em bebes e crianças usando a biometria.

**Comentários**

____________________________________________________________________________________________________________________________________________________________________________________________________________________________________________________________________________________________________________________________________________________________________________________________________________________________________________________________________________________________________________________________________________________________________________________________________________________________________________________________________________________________________________________________________________________________________________________________________________________________________________________________________________________________________________________________________________________________________________________________________________________________________________________________________________________________________________________________________________________________________________________________________________________________________________________
