# Supplementary material for: Acceptability and perceived facilitators and barriers to the usability of biometric registration among infants and children in Manhiça district, Mozambique: A qualitative study
Source: PLoS One. 2021 Dec 17;16(12):e0260631. doi: 10.1371/journal.pone.0260631 (PMC8683034; doi:10.1371/journal.pone.0260631)
Supplement: S3 Appendix — (DOC) [file pone.0260631.s003.doc]

**Department of Social Sciences at CISM**

Biometric data collection in Mozambican infants and children: Evaluation of an Infant and Child Biometric

Prototype to accurately assess unique identity

Short Title:

A Study to Determine Suitability and Stability of Biometrics in Neonates, Infants and Children (BioNIC)

in Manhiça District, Mozambique

Focus Group Discussion Guide (FGDs)

caregivers of children not enrolled in the study

My name is___________________ from Centro de Investigação em Saúde da Manhiça, and I would like to welcome you to this discussion. I will be the facilitator of this FGD and Mr/Mrs _______________ also from Centro de Investigação em Saúde da Manhiça who will take notes and record this discussion with your permission.

**Propósito**

This Focus Group Discussion aims to collect data that will allow us to assess the acceptability of caregivers of children aged 0 to 4 years concerning the use of mobile devices for taking photographs of ears, palms and feet of your children to allow them to accurately identified when they go to the health facility without the child's ID card. Your participation and contribution are important. You were invited to participate in this FGDs because you can provide information that will allow us to assess the acceptability of the biometric prototype and identify facilitators and barriers for its use. The FGDs will last 60 to 90 minutes.

**Basic rules**

We will be recording this FGDs to ensure that none of the answers you give are lost. My colleague will also be taking notes during the discussion. All registered information will be kept confidential and will not be identified by your name. You can choose not to respond at any time.

In order to promote group cohesion and give everyone an equal opportunity to speak, we will follow the following group rules:

All participants will have the opportunity to respond if they wish;

• All participants will wait their turn to speak;

• All participants will respect each other's point of view;

| 1. *Demographics* ***of each participant***  | Nr | Sex | Age | Marital status | Level of education | Able to read? | Able to write? | What is your main occupation? | Religion? | Neighbourhood of residence/ Village | How old is your last born child? | | --- | --- | --- | --- | --- | --- | --- | --- | --- | --- | --- | | 1 |  |  |  |  |  |  |  |  |  |  | | 2 |  |  |  |  |  |  |  |  |  |  | | 3 |  |  |  |  |  |  |  |  |  |  | | 4 |  |  |  |  |  |  |  |  |  |  | | 5 |  |  |  |  |  |  |  |  |  |  | | 6 |  |  |  |  |  |  |  |  |  |  | | 7 |  |  |  |  |  |  |  |  |  |  | | 8 |  |  |  |  |  |  |  |  |  |  | | 9 |  |  |  |  |  |  |  |  |  |  | | 10 |  |  |  |  |  |  |  |  |  |  | | 11 |  |  |  |  |  |  |  |  |  |  | | 12 |  |  |  |  |  |  |  |  |  |  |  1. **Information about the FGDs** |
| --- | --- | --- | --- | --- | --- | --- | --- | --- | --- | --- | --- | --- | --- | --- | --- | --- | --- | --- | --- | --- | --- | --- | --- | --- | --- | --- | --- | --- | --- | --- | --- | --- | --- | --- | --- | --- | --- | --- | --- | --- | --- | --- | --- | --- | --- | --- | --- | --- | --- | --- | --- | --- | --- | --- | --- | --- | --- | --- | --- | --- | --- | --- | --- | --- | --- | --- | --- | --- | --- | --- | --- | --- | --- | --- | --- | --- | --- | --- | --- | --- | --- | --- | --- | --- | --- | --- | --- | --- | --- | --- | --- | --- | --- | --- | --- | --- | --- | --- | --- | --- | --- | --- | --- | --- | --- | --- | --- | --- | --- | --- | --- | --- | --- | --- | --- | --- | --- | --- | --- | --- | --- | --- | --- | --- | --- | --- | --- | --- | --- | --- | --- | --- | --- | --- | --- | --- | --- | --- | --- | --- | --- | --- | --- |
| **Reference of the file: BioNIC_MZ_F1_DGF_** |
| ID do facilitator: |
| ID do notetaker: |
| Location of the FGDs: |
| Date (dd/mm/yyyy):…………/…………/20………… |
| Initial number of FGD participants: |
| Final number of DGF participants: |
| Start time: |
| End time: |
| In which language (s) was the FGDs conducted? |
| Was the FGDs audio-recorded? |
| Brief Description of Participants: |
| Result of the FGDs: |__|Complete |__|Incomplete  Reasons: __________________________________________________________________________________________________  _________________________________________________________________________________________________________ |

**3. Questions**

| 1. In your opinion, is it acceptable to take images for research purposes? Why? 2. Do you find it acceptable to use children’s image for identification? 3. What do you think is it dangerous to use children’s image for identification? If not, what would be the advantages? 4. What part of the body is acceptable / unacceptable for biometric? | **Summary** |
| --- | --- |

Thank you very much for your time and for all of the helpful information you have provided”

End of the FGDs!

Final comments:

We are very grateful that you agreed to participate in this important discussion. We know that we wasted your time, but your contribution is very important and will guide us implementing the Infant and Child Biometric Prototype.

Comments

____________________________________________________________________________________________________________________________________________________________________________________________________________________________________________________________________________________________________________________________________________________________________________________________________________________________________________________________________________________________________________________________________________________________________________________________________________________________________________________________________________________________________________________________________________________________________________________________________________________________________________________________________________________________________________________________________________________________________________________________________________________________________________________________________________________________________________________________________________________________________________________________________________________________________________________
