# Supplementary material for: Acceptability and perceived facilitators and barriers to the usability of biometric registration among infants and children in Manhiça district, Mozambique: A qualitative study
Source: PLoS One. 2021 Dec 17;16(12):e0260631. doi: 10.1371/journal.pone.0260631 (PMC8683034; doi:10.1371/journal.pone.0260631)
Supplement: S4 Appendix — (DOC) [file pone.0260631.s004.doc]

**Departamento de ciências sociais do CISM**

Recolha de dados biométricos em bebês e crianças moçambicanas:

Avaliação de aparelho biométrico infantil em fase de testes (protótipo) para aferir com precisão uma única identidade.

Título abreviado:

Um Estudo para Determinar a Adequação e Estabilidade da Biometria em Neonatos, Bebés e Crianças

no Distrito de Manhiça, Moçambique (Projecto BioNIC)

Guião de Discussão em Grupo Focal (DGF

Trabalhadores de campo

Chamo-me ___________________ do Centro de Investigação em Saúde da Manhiça, e gostaria de lhes dar as boas-vindas a esta discussão. Eu serei o facilitador desta DGF e estará comigo o Sr/Sra. _______________ também do Centro de Investigação em Saúde da Manhiça que vai tomar notas e gravar esta discussão mediante a vossa permissão.

**Propósito**

Esta discussão em Grupo Focal (DGF) como objectivo identificar experiências em relação ao uso do aparelho/dispositivo biométrico infantil em fase de teste, barreiras e facilitadores para a aceitabilidade e uso do dispositivo por parte dos inquiridores responsáveis pela recolha de dados das crianças recrutadas para o estudo. A vossa participação e contribuição é importante. Por isso foram convidados para participar desta discussão em grupo focal (DGF) porque podem fornecer informações que nos permitirão avaliar a usabilidade da biometria infantil e identificar facilidades e dificuldades para o seu uso como forma de garantir uma melhor utilização da identificação única de bebés e crianças. A duração desta discussão em grupo focal está prevista para 60 á 90 minutos.

**Regras Básicas**

Estaremos gravando esta discussão em grupo focal para garantir que nenhuma das respostas que vocês derem não se perca. O meu colega redactor estará também a tomar notas durante a discussão. Todas as informações registadas serão mantidas em sigilo e não serão identificados pelo vosso nome. Vocês podem optar por não responder a qualquer momento.

A fim de promover a coesão do grupo e dar a todos oportunidade igual para falar, vamos seguir as seguintes regras do grupo:

• Todos os participantes terão a oportunidade de responder, se o desejarem;

• Todos os participantes vão esperar pela sua vez para falar;

• Todos os participantes vão respeitar o ponto de vista do outro;

1. **Informação demográfica das participantes**

| Nr | Sexo | Idade | Nível de Escolaridade | Local de trabalho afecto | Recolheu dados na US | Recolheu dados nos agregados familiares | Religião |
| --- | --- | --- | --- | --- | --- | --- | --- |
| 1 |  |  |  |  |  |  |  |
| 2 |  |  |  |  |  |  |  |
| 3 |  |  |  |  |  |  |  |
| 4 |  |  |  |  |  |  |  |
| 5 |  |  |  |  |  |  |  |
| 6 |  |  |  |  |  |  |  |
| 7 |  |  |  |  |  |  |  |
| 8 |  |  |  |  |  |  |  |
| 9 |  |  |  |  |  |  |  |
| 10 |  |  |  |  |  |  |  |
| 11 |  |  |  |  |  |  |  |
| 12 |  |  |  |  |  |  |  |
|  | | | | | | | |
| 1. **Informações sobre a ocorrência da entrevista** | | | | | | | |
| **Referência do ficheiro: BioNIC_MZ_F1_DGF_** | | | | | | | |
| ID do facilitador: | | | | | | | |
| ID do redactor: | | | | | | | |
| Local da realização da DGF: | | | | | | | |
| Data da realização da DGF: | | | | | | | |
| Número inicial dos participantes da DGF: | | | | | | | |
| Número final dos participantes da DGF: | | | | | | | |
| Hora do início da DGF: | | | | | | | |
| Hora final da DGF: | | | | | | | |
| Língua (s) falada (s) durante a entrevista: | | | | | | | |
| A DGF foi gravada: | | | | | | | |
| Breve Descrição dos Participantes: | | | | | | | |
| Resultado da DGF: |__|Completo |__|Incompleto  Razões: __________________________________________________________________________________________________  _________________________________________________________________________________________________________ | | | | | | | |

3. Perguntas para as participantes

| 1. Qual é a sua opinião em relação ao estudo BioNic? 2. Qual é a sua avaliação em relação ao uso do dispositivo biométrico? 3. Qual é a modalidade que foi mais fácil de capturar? 4. Qual é a modalidade que levou menos tempo para capturar? 5. Em que idade (s) a captura de dados é mais fácil? 6. Que dispositivo móvel foi o mais fácil para captura de dados? 7. Como correu o processo de recrutamento das crianças. 8. Quais foram as reações dos pais ou tutores quando foram abordados para fazerem parte do estudo. 9. Os pais ou responsáveis das crianças fizeram perguntas em relação ao processo de recolha de dados? Quais foram essas perguntas? 10. Qual foi o nível de satisfação dos pais ou tutores das crianças? 11. Na sua opinião o que levou os pais ou tutores a aceitarem participar do estudo? 12. Caso tenham registados recusas no processo de recrutamento, quais acham que podem ter sido as causas que levaram os pais ou tutores a recusarem? | **RESUMO** |
| --- | --- |

**Fim da DGF!**

**Comentários finais:**

Estamos muito gratos por vocês terem concordado em participar desta importante discussão. Sabemos que ocupamos o vosso precioso tempo. As vossas contribuições foram muito importantes e irão nos orientar na implementação da identificação única em bebes e crianças usando a biometria.

**Comentários**

____________________________________________________________________________________________________________________________________________________________________________________________________________________________________________________________________________________________________________________________________________________________________________________________________________________________________________________________________________________________________________________________________________________________________________________________________________________________________________________________________________________________________________________________________________________________________________________________________________________________________________________________________________________________________________________________________________________________________________________________________________________________________________________________________________________________________________________________________________________________________________________________________________________________________________________
