# Supplementary material for: Acceptability and perceived facilitators and barriers to the usability of biometric registration among infants and children in Manhiça district, Mozambique: A qualitative study
Source: PLoS One. 2021 Dec 17;16(12):e0260631. doi: 10.1371/journal.pone.0260631 (PMC8683034; doi:10.1371/journal.pone.0260631)
Supplement: S5 Appendix — (PDF) [file pone.0260631.s005.PDF]

Table: Consolidated criteria for reporting qualitative studies (COREQ): 32-item checklist

**No**	**Item**	**Guide question/description**	**Answer**
	Domain 1: Research team and Reflexivity		
	Personal characteristics		
1	Interview/facilitators	Which author/s coordinated the interview or focus groups?	Olga Cambaco, Célia Chaúque and Estevão Mucavele
2	Credentials	What were the researcher's credentials?	Graduation (Bsc degree)
3	Occupation	What was their occupation at the time of the study	2 (OC and EM) Junior Researchers and 1 (CC) MPH trainee
4	Gender	Was the researcher male or female?	2 Females and 1 Male
5	Experience and training	What experience and training did the researcher have?	Qualitative analysis, ICH Good Clinical Practices, Training in qualitative data collection tools, Training in Qualitative Data Analysis using Nvivo 11, Training in Implementation Science, Gender training, English Course and REDcap Training for Data Management
	Relationship with participants		
6	Relationship established	Was a relationship established prior to study the commencement?	Yes.
7	Participant knowledge of the interviewer	What did participants know about the researcher	Reasons for conducting the study and the institution conducting the researcher.
8	Interviewer characteristics	What characteristics were reported about the interviewer/facilitator?	Bias regarding participant selection.

	Domain two: Study design		
9	Methodological orientation and theory	What methodological orientation was stated to underpin the study?	Phenomenology Theory
	Participant Selection		
10	Sampling	How were participants selected?	Purposive sampling
11	Methodology of approach	How were participants approached?	Face-to-face
12	Sample size	How many participants were in the study?	65 participants considered eligible for data analysis: 52 caregivers; 5 healthcare providers and 8 data collectors.
13	No-participation	How many people refused to participate or dropped out? Reasons	None
	Setting		
14	Setting of data collection	Where was the data collected?	Focus group with caregivers took place in the community and healthcare facilities; healthcare providers were interviewed in their working place – healthcare facility, and focus group discussion with data collectors took place at Manhiça Health Research centre (CISM)
15	Presence of non-participants	Was anyone else present beside the participant and the researcher?	No.
16	Description of sampling	What are the important characteristics of the sampling?	Age, education, occupation, being mother of child aged between zero and five years old.
	Data collection		
17	Interview guide	Where questions, prompts, guides provided by the authors? Was it pilot tested?	Yes, the question guides were designed and pilot tested to participants with similar characteristics of the study group in a different setting of the main study area. The final guide was revised, and approved by all authors.

18	Repeated interviews	Were repeated interviews carried out?	Focus group discussion with caregivers and interviews with healthcare providers were not repeated. But focus group discussion with data collectors were repeated.
19	Audio/visual recording	Did the researcher use audio or visual recording to collect the data?	Yes, audio recording was used to collect data with all participants.
20	Field notes	Were field notes made during or after interview or focus group	Yes, field notes were made during interviews and focus group discussions
21	Duration	What was the duration of interview or focus group?	Individual interviews among healthcare providers lasted between 16 minutes and 40 minutes;<br>Focus group discussion with caregivers and data collectors lasted between 80 and 120 minutes.
22	Data saturation	Yes, data saturation was discussed	Yes, data saturation was discussed during data collection.
23	Transcript returned	Were transcript returned for participants for comments and or/comments	No. Transcriptions were not returned to each individual participant. But, meetings were held with some participants to discuss the preliminary findings of data analysis.
	Domain 3: Analysis and findings		
	Data analysis		
24	Number of data coders	How many data coders coded the data?	3
25	Description of the coding tree	Did authors provide the description of coding tree?	Yes.
26	Derivation of themes	Were themes identified in advance or derived from the data?	Some themes were identified in advance. However, the analysis used open coding, which enabled the identification of the others themes and subthemes emerging from the data.
27	Software	What software, if applicable, was used to manage the data?	NVivo

28	Participants checking	Did participants provide feedback on the findings?	Yes, through seminars organized in the community to discuss the preliminary findings.
	Reporting		
29	Quotations presented	Where participants quotations presented to illustrate the themes/findings/Was each quotation identified?	Yes. However, each quotation was labelled two general social characteristics. This method aimed at preserving the identity of the participants.
30	Data and finding consistence	Was there consistence between the data presented and the findings?	Yes.
31	Clarity of major themes	Were major themes clearly presented in the findings?	Yes.
32	Clarity of minor themes	Is there a description of diverse cases or discussion of minor themes?	Yes.
